# Detecting Older Adults’ Behavior Changes During Adverse External Events Using Ambient Sensing: Longitudinal Observational Study

**DOI:** 10.2196/69052

**Published:** 2025-05-01

**Authors:** Roschelle Fritz, Diane Cook

**Affiliations:** 1 Betty Irene Moore School of Nursing, University of California Davis Health, 4610 X St, Sacramento, CA, 95817, United States, 1 9167344349; 2 School of Electrical Engineering and Computer Science, Washington State University, Pullman, WA, United States

**Keywords:** internet of things, digital phenotyping, chronic disease, COVID-19, air pollution

## Abstract

**Background:**

Older adults manage multiple impacts on health, including chronic conditions and adverse external events. Smart homes are positioned to have a positive impact on older adults’ health by (1) allowing new understandings of behavior change so risks associated with external events can be assessed, (2) quantifying the impact of social determinants on health, and (3) designing interventions that respond appropriately to detected behavior changes. Information derived from smart home sensors can provide objective data about behavior changes to support a learning health care system. In this paper, we introduce a smart home capable of detecting behavior changes that occur during adverse external events like pandemics and wildfires.

**Objective:**

Examine digital markers collected before and during 2 events (the COVID-19 pandemic and wildfires) to determine whether clinically relevant behavior changes can be observed and targeted upstream interventions suggested.

**Methods:**

Secondary analysis of historic ambient sensor data collected on 39 adults managing one or more chronic conditions was performed. Interrupted time series analysis was used to extract behavior markers related to external events. Comparisons were made to examine differences between exposures using machine learning classifiers.

**Results:**

Behavior changes were detected for 2 adverse external events (the COVID-19 pandemic and wildfire smoke) initially and over time. However, the direction and magnitude of change differed between participants and events. Significant pandemic-related behavior changes ranked by impact included a decrease in time (3.8 hours/day) spent out of home, an increase in restless sleep (946.74%), and a decrease in indoor activity (38.89%). Although participants exhibited less restless sleep during exposure to wildfire smoke (120%), they also decreased their indoor activity (114.29%). Sleep duration trended downward during the pandemic shutdown. Time out of home and sleep duration gradually decreased while exposed to wildfire smoke. Behavior trends differed across exposures. In total, two key discoveries were made: (1) using retrospective analysis, the smart home was capable of detecting behavior changes related to 2 external events; and (2) older adults’ sleep efficiency, time out of home, and overall activity levels changed while experiencing external events. These behavior markers can inform future sensor-based monitoring research and clinical application.

**Conclusions:**

Sensor-based findings could support individualized interventions aimed at sustaining the health of older adults during events like pandemics and wildfires. Creating care plans that directly respond to sensor-derived health information, like adding guided indoor exercise, web-based socialization sessions, and mental health–promoting activities, would have practical impacts on wellness. The smart home’s novel, evidence-based information could inform future management of chronic conditions, allowing nurses to understand patients’ health-related behaviors between the care points so timely, individualized interventions are possible.

## Introduction

### Background and Significance

The older adult population is rapidly growing, with 95% of persons aged 60+ managing a chronic condition and 80% managing 2 or more [1]. These same older adults are also experiencing more external events with the potential to impact self-management of their chronic conditions, such as wildfire smoke and COVID-19. Standard approaches to managing chronic conditions do not typically account for the impact of external events. Innovative technological approaches that (1) operate across diverse settings, (2) support a learning health care system, and (3) incorporate a social determinants of health (SDOH) lens are essential to enhance self-management of health conditions and support aging in place. Vulnerable populations often experience greater effects of external events due to reduced resources [2-4]. Besides managing chronic health conditions, 80% of U.S. older adults face income insecurity [5], reducing their capacity for self-management of the impacts of external events. For example, they may not be able to travel to a location with better air quality during a wildfire or to less crowded spaces during a pandemic.

Identifying specific behavior changes in response to external events presents opportunities for early nursing interventions. If behavior and health changes emanating from such events can be detected and understood, then smart homes could support automated upstream interventions like personalized activity cues and health education. Our prior work and that of others noted changes in health behavior that occurred during one such external event, the COVID-19 pandemic [6,7]. Similarly, people experienced changes and complications during wildfire season. The risk and extent of wildfires in the Pacific Northwest have doubled in recent years [8]. These increasingly large and intense wildfires are causing a spike in unhealthy pollutants, posing health risks to millions of people, and confining many older adults to their homes each summer [9]. Prior research observed that while particulate matter (PM2.5) and ozone (O_3_) were raised primarily outdoors, acetonitrile and benzene were also elevated indoors during fires [10-14]. Evidence is mounting that neighborhood-level exposure to particulate matter adds to the risk of health decline [15,16], crossing the blood-brain barrier and causing neural inflammation [17].

This work is based on larger studies in which we model behavior from passive sensors to detect and react to changes in physiological and cognitive health. Because sensors were placed in participant homes before events such as wildfires and the pandemic shutdown took place, we monitored behavior before and during these events. Participants reported behavior changes and health issues that were related to these events. The goal of this work was to analyze sensor data to detect, quantify, and analyze these changes. Our data analysis hypotheses were:

1. Changes in behavior will be observed between nonevent and event time periods.
2. The amount and type of behavior changes will differ based on parameters such as prior health conditions, age, and demographics.
3. Initial behavioral changes in response to the event may differ from those that emerge as the event persists.

The intended outcome of the work is to suggest possible interventions that prevent unhealthy behavior changes and mitigate the health impact of such external events.

### Prior Work

Researchers have observed changes in health during events that force more indoor activity, such as the COVID-19 lockdown and wildfire-driven poor air quality. For example, Krendl et al [18] and Burke et al [19] found these events to be associated with higher amounts of depression and loneliness based on individual self-report. However, Balki et al [20] noted that some of these health impacts are mitigated by individual factors such as gender and education. These types of events also spark changes in behavior. These include changes in nighttime and daytime sleep patterns, as observed by Gupta et al [21] Salfi et al [22] found that for some groups these behaviors change at first and then ease back to pre-event behavior, while other groups experience greater behavior change as the event continues. Their study confirmed the role of social determinants of health on behavior change during the pandemic.

While passively monitoring and modeling human behavior has become achievable with ambient and wearable sensors [23,24], little work has used sensors to capture behavior patterns and changes during external events like a pandemic or wildfire smoke to determine health impact and support. Collecting such data was particularly challenging during the pandemic when study participants could not be visited in person. However, a few projects were successful in assembling and assessing related data. In particular, Rajkumar et al [25] plotted movement levels inside 3 homes to visualize changes in the areas of the home that were frequented based on motion sensor reports. Leese et al [26] monitored driving and computer use over 5 months to quantify the decrease in driving distance and increase in time spent on the computer. The work reported in this study is based on longitudinal data from multiple studies collected in the homes of older adults with significant health risks before and during external events. This offers a unique opportunity to analyze behavior change from passive, continuous sensor observations.

Table 1 positions this study in comparison with prior work. As shown in the table, researchers have investigated the impacts of wildfire smoke and COVID-19 lockdowns on behavior, though none of these have investigated multiple events. Most of the study mechanisms rely on self-reports provided through digital surveys. One exception is the work of Ceolotto et al [27], who analyzed wastewater during the pandemic to quantify changes in the use of prescription drugs, nicotine, and alcohol. The work that is closest to our study is that of Rajkumar et al [25], which analyzed data from motion sensors to visualize social isolation for 3 homes during the COVID-19 pandemic. In comparison with these prior studies, we use longitudinal sensor data to compare pre-event and mid-event behavior. Performing this analysis for multiple event types (wildfire smoke events and pandemic lockdown events) facilitates comparison of behavior impact between diverse adverse external events.

**Table 1.** Summary of related studies.

Study	Event	Behavior	Collection mechanism
Stewart [28]	Wildfire smoke	Personal perceptions	Survey, air monitors
Burke [19]	Wildfire smoke	Depression, time at home	Survey, phone or web-based activity
Hu [29]	COVID-19	Smoking, alcohol, nutrition, sleep	Survey
Salfi [22]	COVID-19	Sleep	Survey
Gupta [21]	COVID-19	Sleep	Survey
Krendl [18]	COVID-19	Depression	Survey, social network
Leese [26]	COVID-19	Car, computer use	Survey, car computer
Ceolotto [27]	COVID-19	Medicine, caffeine, nicotine use	Wastewater
Rajkumar [25]	COVID-19	Isolation	Motion sensors
This paper	Wildfire smoke, COVID-19	Sleep, time out of home, activity level	Motion sensors, door sensors, weekly telehealth with self-report or nurse observation

## Methods

### Participants

Participants were community-dwelling adults (n=39) recruited from the Pacific Northwest region of the United States through advertising and involvement in prior studies. Inclusion criteria were living independently in their own home, having an internet connection, and the ability to communicate in English. Of the participants, 37 were older adults (70+ years), and 2 were healthy younger adults (<35 years) included for comparison. Participant characteristics are summarized in Table 2.

**Table 2.** Summary of participant information.

Event and age		Age (years), mean (SD)	Gender	Education (years), mean (SD)	Conditions
COVID (n=13)					
	<35 years	23.5 (4.95)	1 male; 1 female	19.50 (2.12)	Healthy
	70+ years	83.82 (6.11)	2 male; 9 female	16.75 (1.83)	COPD^a^ (1), asthma (1), diabetes mellitus (2), CHF^b^/AFib^c^ (4), coronary artery disease (2), HTN^d^ (5), arthritis (3), stroke (2), obesity (2), macular degeneration (3)
Smoke (n=28)					
	70+ years	91.10 (5.89)	7 male; 13 female; 8 not reported	17.50 (2.38)	Mild cognitive impairment (3), HTN (1), COPD (1), cancer (1)

a COPD: chronic obstructive pulmonary disorder.

b CHF: congestive heart failure.

c AFib: atrial fibrillation.

d HTN: hypertension.

### Data Collection

#### Overview

Ambient sensors were placed in each participant’s home and continuously collected data for a minimum of 1 year while residents performed their regular daily routines. In total, 2 types of sensor units were used: passive infrared motion detectors combined with ambient light sensors were placed on ceilings in each functional area (2‐4 sensors per room) to monitor movement and light levels. Additionally, magnetic units with door sensors and ambient temperature sensors were placed on external doors and kitchen or bathroom cabinets to monitor door usage and temperature changes.

Registered nurses conducted weekly telehealth visits for the duration of the study. Participants were asked, “How has your health been over the last week? Did you experience any changes in your health? If so, what changed?” Narrative summaries were recorded each week of participants’ self-reported health status and nurses’ observations. Blood pressure, heart rate, oxygen saturation, and pain level were also recorded weekly. These data informed the machine learning analytics.

#### Event Groups

For this data analysis, we selected homes with 1 resident and no pets to focus on behavior change for 1 participant in each home and reduce noise. When behavior is analyzed in homes with multiple residents, the sensor data reflect the collective behavior of everybody in the home. Without attributing behavior to specific residents in such a group setting, direct comparisons cannot be easily made between single-resident and multi-resident homes. Additionally, we restricted our analysis to homes that included multiple days of data collection before the events and during events. The homes were grouped based on 2 event types: 1 set of 13 homes (COVID) collected sensor data before and during the COVID-19 pandemic lockdowns. Reflecting a second event, a set of 28 homes (smoke) collected data before and during times with poor air quality due to wildfire smoke.

In the COVID group, we analyzed data from March 17, 2020, through May 21, 2020, during which the region followed a stay-at-home protocol. For baseline comparison, we analyzed an equivalent number of season-matched days from the previous year. In the smoke group, we analyzed time periods containing at least 2 consecutive days with an air quality index >100 (indicating the air quality is unhealthy or hazardous) and an equivalent number of baseline days with air quality index ≤50 (indicating good air quality) during the same month. None of the COVID and smoke dates overlapped. In total, 2 of the homes collected data in both conditions and are included in both analyses. Additionally, we removed dates in which the participant was outside the home more than half the day. Sensor performance was routinely monitored, and sensors were removed from analysis if their performance was not reliable. In a few instances, all sensors failed to report information for a given date. When this occurred, we removed the date from consideration. In total, we analyzed 1990 days for the COVID group and 1568 days for the smoke group.

#### Digital Behavior Markers

We defined a collection of digital markers that could be extracted from ambient sensor readings and used to describe daily behavior. The markers describe sleep, time out of the home, and activity level. These behavioral categories are reported to be influenced by poor air quality and pandemic shutdowns [21,22,30-33]. These behaviors in turn impact physiological and psychological well-being, particularly for individuals managing chronic health conditions [34-39].

In this analysis, nighttime sleep is detected between 9pm and 7am when motion sensor readings are ≥5 minutes apart and the most-recently sensed location of the resident is the bedroom. If there are >2 contiguous motion sensor readings outside the bedroom, the state is considered awake. If the awake state is surrounded by sleep in the same evening, the awake state is a sleep interruption rather than the end of the night’s sleep.

The motion sensors combined with the door sensors define when a participant is out of the home. Specifically, if the person’s most recent state was awake, >20 minutes elapsed between motion sensor readings, and the most recent sensor readings are from an external door or door area, the person is considered out of the home until >2 sensor readings occur inside the home.

Finally, activity level is estimated by the normalized count of motion sensor readings occurring when the participant is home. The number of sensors inside a person’s home varies depending on the size of the residence and the number of rooms. To accommodate the resulting differences in sensor quantity and density, all markers that rely on a motion sensor count are normalized with a standard scaler based on each person’s daily motion sensor counts.

**Figure 1.**
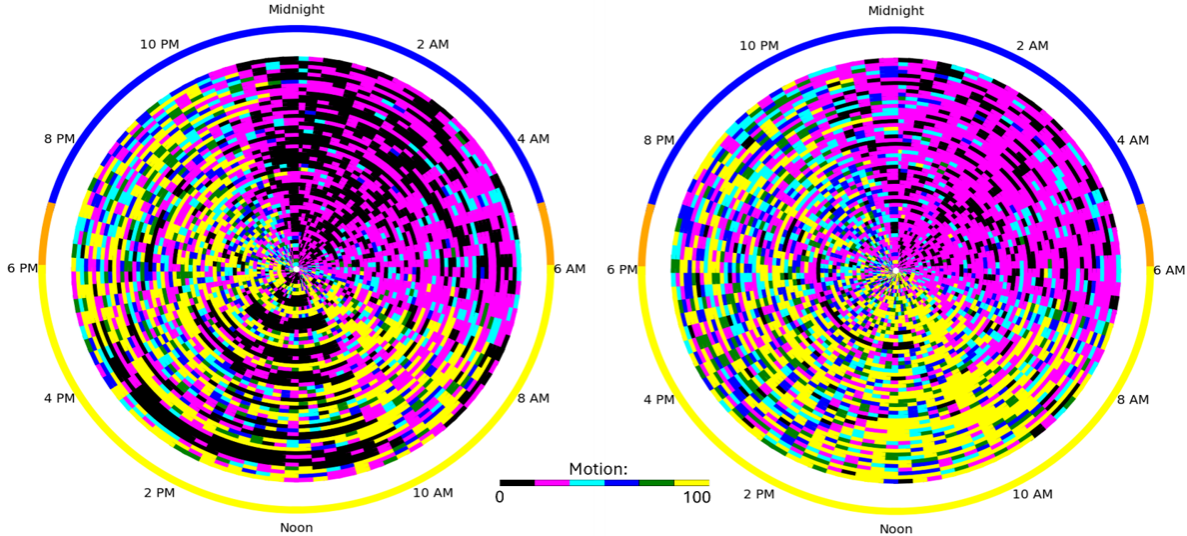
Radial plots for 1 home showing activity level by time of day; 1 ring per day. (Left) Prepandemic behavior and (right) pandemic shutdown behavior. Colors indicate an increasing amount of motion from black (little or no motion) to yellow.

Figure 1 shows a plot of motion observed in 1 home during the prepandemic shutdown (left) and during the pandemic shutdown (right). In this plot, where each ring signifies a distinct day, we can observe some of the changes that were sensed between these times. Black regions indicate a lack of motion sensor readings. Before the shutdowns, black occurred throughout the day when the resident was out of the home and throughout nighttime sleep. During the shutdown, the number of daytime outings is greatly reduced. Furthermore, while sleep can still be detected at night, the person is more restless at night, with the black regions being replaced by more magenta and cyan periods. To analyze changes in these characterizing behaviors, we define the digital markers for each day as follows: sleep duration = the time spent in bed between the night’s first and last detected sleep (Textbox 1).

Textbox 1. **Definition for digital markers for each day**

- Sleep duration: the time spent in bed between the night’s first and last detected sleep.
- Sleep efficiency: following recommendations by the National Science Foundation [40], this is defined as the nighttime ratio of sleep time to time spent in bed.
- Sleep restlessness (normalized): the number of motion sensor readings that are generated while the person is asleep.
- Time out: time spent outside the home.
- Activity level (normalized): the number of motion sensor readings generated divided by the time spent at home.

### Data Analysis

We apply an interrupted time series (ITS) analysis to assess the impact of an event that disrupts an ongoing time series [41]. Behavior markers *X_t_* are collected for each day, *t*. This marker sequence forms a time series that is interrupted by an event, *E*:


(1) $$X_{-3},X_{-2},X_{-1},\left(E\right),X_{+1},X_{+2},X_{+3}$$

ITS allows us to perform a counterfactual analysis, estimating what would have happened to a person’s behavior if the event had not occurred. In ITS, this is done by projecting the pre-event behavior trend (the counterfactual) into the postintervention trend. We perform segmented regression analysis to examine changes in level and trend over time, both before and during the event, allowing us to estimate its effect. We estimate the trend before the event, the immediate impact of the event, and the trend after the event, controlling for age, gender, and education. Where the results of the counterfactual analysis are not consistent across participants, we generate participant phenotypes using k-means clustering (k=3) and report statistics for individual groups.

Additionally, we use a machine learning classifier to predict if a set of behavior markers belongs to the nonevent or event group. This analysis captures nonlinear relationships and complex interactions between the variables to determine whether the event caused clear, measurable differences between the periods. For this analysis, we employ a random forest classifier with 100 trees and report results based on 5-fold cross-validation. We also use the classifier to quantify and rank the markers for their importance in distinguishing between nonevent periods, COVID periods, and wildfire smoke periods. Using random forests to promote interpretability of machine learning algorithms is a highlight of the method that has been explored by other researchers to predict events such as hospitalization among older adults [42]. Features are ranked by the Gini impurity (GI) measure, which guides the construction of the decision trees in the random forest.

### Ethical Considerations

This study was approved by Washington State University Institutional Review Board (IRB#15412). Studies from which data were collected for this secondary analysis were also reviewed and approved by the Institutional Review Board at Washington State University. All data were anonymized before performing analyses. Participants voluntarily consented after receiving information about the study and verbalizing their understanding. Participants’ data were confidentially linked during their participation in the study and unlinked upon completion. After completing the study, participants received a US $250 gift card.

## Results

**Figure 2.**
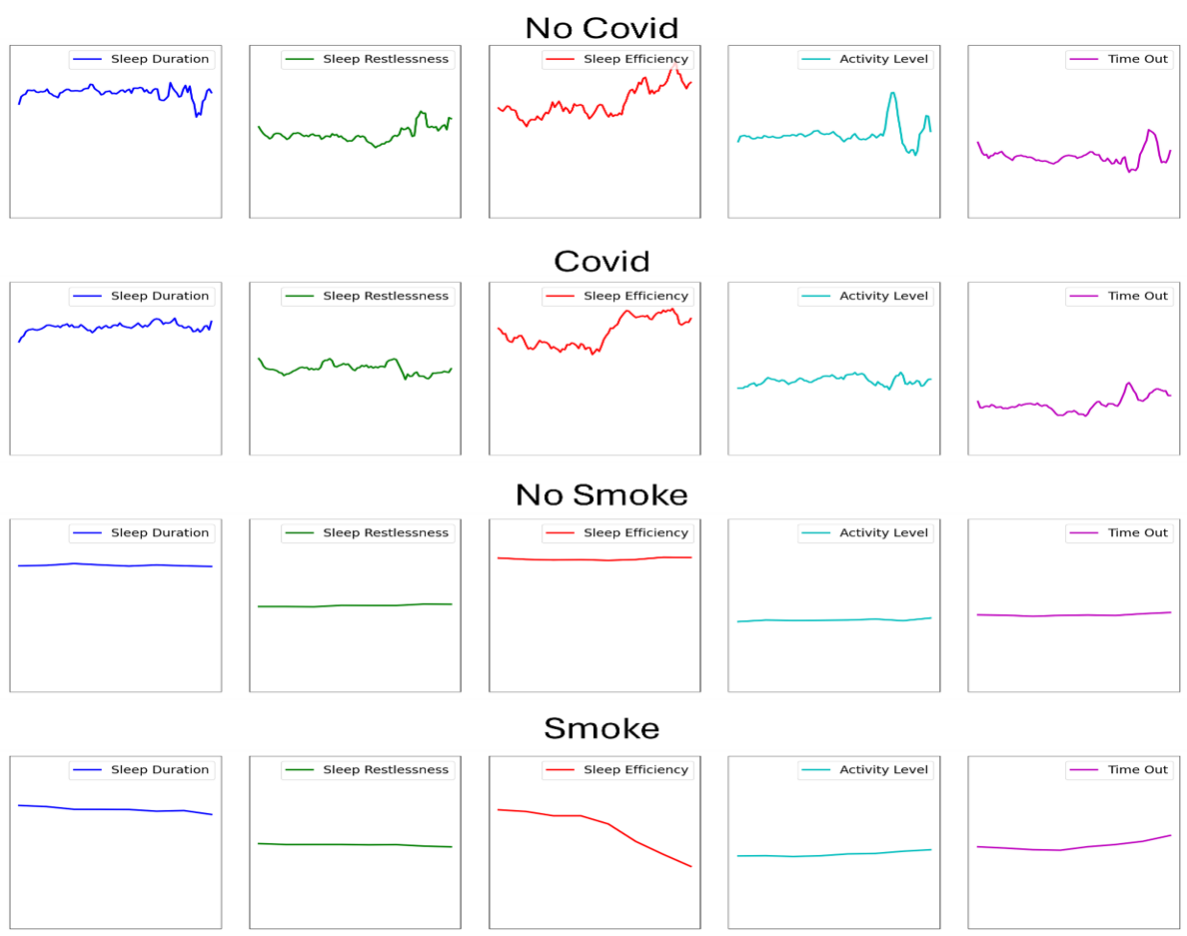
Trend lines for the markers across event occurrences and participants. The x-axis of each plot indicates the date in the time series and the y-axis indicates the mean value of the marker at that point in the sequence.

Tables 3 and 4 summarize the ITS analysis results for COVID-19 and wildfire smoke events, showing differences in the type, degree, and direction of behavior changes between the 2 events. Similarly, Figure 2 shows the values of the markers as a function of the day in the time series before and during each event, though these values are aggregated over the entire sample. Before the pandemic shutdown, behavior markers remained stable, with changes of less than 0.08%. In contrast, the immediate impact of the event was more pronounced. Sleep duration increased slightly, while sleep restlessness showed a significant rise of 946.74%. Sleep efficiency remained relatively constant, but indoor activity decreased by 38.89%. As expected, time out of the home reflected the largest change, decreasing from 5.97 hours daily to an average of 2.17 hours, a statistically significant reduction.

**Table 3.** Results of interrupted time series analysis applied to daily behavior markers for the COVID-19 event (n=13). Model strength is reported as *F* test scores; sleep duration and time out of home are reported in seconds. Results are summarized for pre-event baseline (initial), trend before the event occurred (pre-event trend), impact on the first day of the event (immediate impact), and trend from the beginning to the end of the monitored event (long-term trend). Results are further broken down by gender.

Variable		*F* test		Initial		Pre-event trend		Immediate impact		Long-term trend	
		*F* score (*df*)	*P* value	Value	*P* value	Value	*P* value	Value	*P* value	Value	*P* value
Sleep duration (seconds)											
	Total	11.96 (3, 9)	<.001	28,900	<.001	4.00	<.001	22.00	.98	–1.96	<.001
	Female	12.84 (3, 9)	<.001	29,070	<.001	4.24	<.001	–451.64	.69	–0.47	<.001
	Male	13.78 (3, 9)	<.001	29,030	<.001	0.24	.86	–476.21	.74	0.48	.87
Restlessness^a^											
	Total	6.58 (3, 9)	<.001	–0.09	.047	–6.65e-05	<.001	0.762	<.001	0.00	<.001
	Female	10.59 (3, 9)	<.001	–0.18	<.001	–7.00e-05	<.001	1.11	<.001	–2.00e-03	.28
	Male	2.064 (3, 9)	.10	0.14	.13	–5.00-04	.09	0.00	.99	3.00e-04	.61
Sleep efficiency^b^											
	Total	45.93 (3, 9)	<.001	0.79	<.001	0.00	<.001	0.00	.97	0.00	.75
	Female	53.37 (3, 9)	<.001	0.78	<.001	2.00e-04	<.001	0.03	.38	–7.00e-05	.28
	Male	49.83 (3, 9)	<.001	0.87	<.001	8.35e-05	<.001	–0.14	<.001	2.00e-04	<.001
Activity level^c^											
	Total	25.17 (3, 9)	<.001	0.54	<.001	0.00	<.001	–0.21	.27	0.00	.88
	Female	12.37 (3, 9)	<.001	0.52	<.001	–6.07	.001	–0.61	.002	1.10e-03	.003
	Male	1.534 (3, 9)	.20	0.47	<.001	–4.00e-04	.18	0.67	.04	0.00	.001
Time out (seconds)											
	Total	14.55 (3, 9)	<.001	21,500	<.001	–6.00	<.001	–13,700	<.001	26.00	<.001
	Female	20.84 (3, 9)	<.001	22,090	<.001	–9.88	<.001	–5330	.03	8.05	.07
	Male	3.77 (3, 9)	.01	19,090	<.001	18.36	<.001	–30,130	<.001	44.31	<.001

a Number of motion sensor readings that are generated while the person is asleep.

b Nighttime ratio of sleep time to time spent in bed.

c Number of motion sensor readings generated divided by the time spent at home.

**Table 4.** Results of interrupted time series analysis applied to daily behavior markers for the wildfire smoke event (n=30). Model strength is reported as *F* test scores; sleep duration and time out of home are reported in seconds. Results are summarized for pre-event baseline (initial), trend before the event occurred (pre-event trend), impact on the first day of the event (immediate impact), and trend from the beginning to the end of the monitored event (long-term trend). Results are further broken down by gender.

Variable	*F* test		Initial		Pre-event trend		Immediate impact		Long-term trend	
	*F* score (*df*)	*P* value	Value	*P* value	Value	*P* value	Value	*P* value	Value	*P* value
Sleep duration (seconds)										
Total	46.51 (3, 24)	<.001	30,860	<.001	–0.17	.69	–1652.49	.28	–1.95	.01
Female	24.78 (3, 24)	<.001	30,790	<.001	–0.02	.98	–977.25	.58	–1.48	.14
Male	33.15 (3, 24)	<.001	28,320	<.001	0.77	.20	–1214.66	.52	–2.79	<.001
Restlessness^a^										
Total	3.83 (3, 24)	.01	0.15	.06	0.00	.001	–0.18	.29	0.00	.13
Female	2.06 (3, 24)	.10	0.15	.17	–2.00e-04	.03	–0.11	.61	9.00e-05	.47
Male	4.04 (3, 24)	.008	0.11	.14	–1.00e-04	<.001	–0.16	.24	1.00e-04	.07
Sleep efficiency^b^										
Total	123.50 (3, 24)	<.001	0.92	<.001	0.00	.64	–0.06	.13	0.00	<.001
Female	49.83 (3, 24)	<.001	0.92	<.001	4.56e-06	.77	–0.02	.60	–8.53e-05	<.001
Male	71.55 (3, 24)	<.001	0.87	<.001	4.11e-06	.82	–0.06	.30	–1.00e-04	<.001
Activity level^c^										
Total	0.46 (3, 24)	.71	0.07	.46	0.00	.94	–0.08	.65	0.00	.49
Female	1.53 (3, 24)	.21	–0.12	.30	2.00e-04	.04	0.19	.39	–1.00e-04	.29
Male	1.41 (3, 24)	.24	0.15	.26	–8.22e-05	.26	–0.26	.27	2.00e-04	.10
Time out (seconds)										
Total	1.48 (3, 24)	.22	26,180	<.001	0.86	.25	1969	.46	–2.00	.16
Female	3.77 (3, 24)	.01	27,640	<.001	0.34	.77	1916	.56	0.84	.65
Male	7.89 (3, 24)	<.001	22,280	<.001	2.78	.007	4676	.15	–5.10	.002

a Number of motion sensor readings that are generated while the person is asleep

b Nighttime ratio of sleep time to time spent in bed.

c Number of motion sensor readings generated divided by the time spent at home.

The overall impact of events is relatively consistent when we consider participants by gender, though a few differences are noted. Specifically, female participants experienced more of a long-term decrease in sleep duration and sleep efficiency during the COVID-19 lockdown. They also decreased their activity level at the beginning of the event, though it did increase as the event continued. Additionally, male participants experienced a greater impact of the event in terms of decreased time out of the home, though this time increased more than for the women as the pandemic continued.

Unlike the COVID-19 shutdown, none of the immediate behavior changes were significant when wildfire smoke began. Participants decreased their sleep duration by 5.36% and sleep efficiency by 6.52%, but sleep appeared to improve in quality, with a 120% decrease in restlessness. Indoor activity decreased by 114.29%, while time out of the home increased by 7.52%, an average of 32.82 additional minutes a day.

Postevent trends also revealed notable differences. During the pandemic, sleep duration initially increased but gradually declined over time, while time out of the home, though initially reduced, gradually rose over the 66-day shutdown. In contrast, during extended periods of wildfire smoke, both “time out of the home” and “nighttime sleep duration” gradually decreased.

Differences between gender subgroups were largest for time spent out of the home. At the beginning of wildfire smoke events, male participants spent more time out of the house. As the poor air quality continued for multiple days, however, this group significantly decreased their time spent out of the home each day.

**Figure 3.**
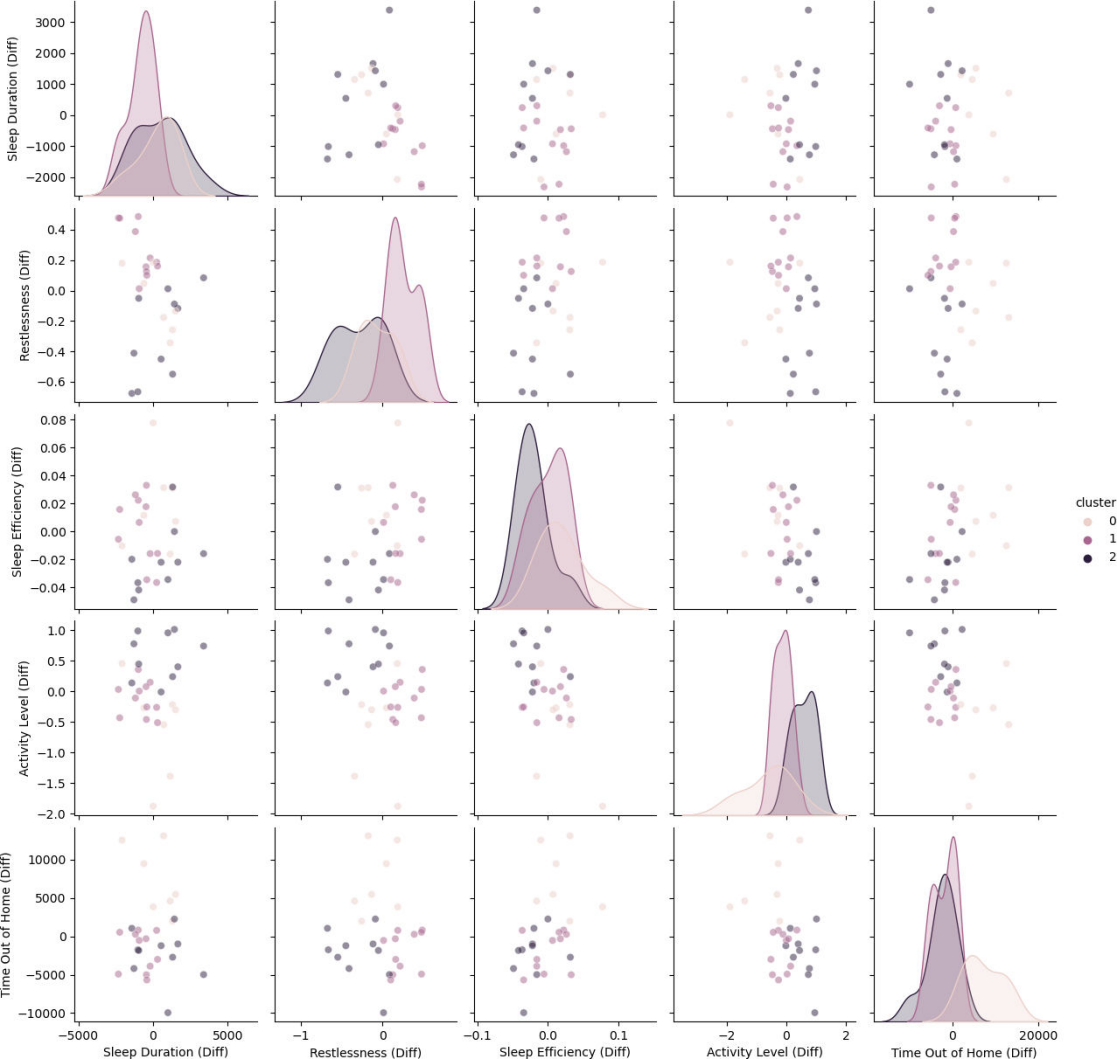
Phenotypes of smoke impact on participant behavior. Plots show the difference of the behavior marker mean for each participant between the event (smoke) period and the nonevent period. Cluster sizes are (cluster 0: n=7, cluster 1: n=11, cluster 2: n=10).

Behavior changes due to wildfire smoke were neither large nor statistically significant. However, some participants reported experiencing health and behavior changes during these times. To determine whether results vary between subgroups, we used k-means clustering (k=3) to identify participant phenotypes. The results, shown in Figure 3, highlight some important differences. Clusters 0 and 2 show minor behavior differences: cluster 0 exhibits a slight increase in time spent out of the home, while cluster 2 shows a slight increase in activity level and decrease in sleep efficiency. In contrast, participants in cluster 2 exhibit more pronounced changes, with a 182.93% increase in sleep restlessness (*P=*.002) and a 176.14% decrease in activity level (*P=*.06).

To assess the predictive nature of event behaviors, we examined the random forest predictions and the results are summarized as follows. Accuracy was 0.68 for classifying a participant as COVID, no COVID, smoke, or no smoke; 0.72 for classifying as positive (COVID or smoke) versus negative (no COVID, no smoke); 0.76 for predicting COVID versus no COVID; and 0.71 for predicting smoke versus no smoke. For comparison, expected accuracy using a random classifier was 0.25 for the 4-class case and 0.50 for the 3 binary classification tasks. Because we analyze an equal number of pre-event and mid-event days, these class distributions are balanced. As a result, we use predictive accuracy as the performance metric. The results indicate that all behavior predictions were significantly more accurate than random guessing (*P*<.001). While the difference in behavior between prepandemic and postpandemic periods was the most predictable, behavior differences between smoke and no-smoke periods were also highly predictive. We ranked the behavior markers by their predictive value for each event. The most predictive marker was time out of the home (GI=.108), followed by sleep restlessness (GI=.078), sleep efficiency (GI=.071), activity level (GI=.068), and sleep duration (GI=.050). Features were ranked in this order for all the prediction tasks.

## Discussion

### Principal Findings

Sensor data represent a new form of “informatics evidence” that supports informatics triage—a future requirement for home-based health technologies. These data provide objective evidence to inform decision support tools and clinical judgments. Aligned with value-based care ideals [43], information derived from smart home sensors can help prevent (re)hospitalizations and reduce unnecessary emergency room visits, promoting overall health and extending independence through health maintenance support. However, for smart home data to meaningfully reflect the impacts of external events on older adults or to predict health risks, we must understand how routine behaviors change with exposure. This study provides evidence that ambient sensing reliably captures exposure-related behaviors. The selected digital markers and analysis offer insight into how exposures are behaviorally expressed when older adults are in their home, where they are arguably their most authentic selves.

Discerning behavior changes by type, degree, and trend is essential. Changes from baseline (nonevent) to new (event) behavior may involve variations in activity frequency, timing, duration, or location. Clinically relevant findings included restlessness during sleep and reduced time spent outside the home, both associated with heightened health risks. Poor sleep quality is associated with increased risk for all-cause cardiovascular mortality [44]. Decreased physical activity and social interaction increase the risk for poor mental health [45], dementia [46], cardiovascular disease, and cancer care outcomes [44]. As a result, clinicians commonly rely on knowledge of such behavior trends for clinical decision-making.

Objective, real-time evidence of key behavior changes creates opportunities for impactful, low-cost interventions, such as activity cueing [47,48], as well as community-level interventions addressing social determinants of health. For example, older Asian immigrants experienced unique needs during the pandemic lockdowns where, besides managing their health, they also managed an associated external event related to Asian hate [49], leading many older Asian Americans to remain at home for safety purposes. Behavior changes detected from the smart home digital markers we illuminate here could assist clinicians and community-based organizations in prioritizing and mobilizing community health workers among their constituents [50]. Indeed, discrimination reported by older Asian Americans during the pandemic resulted in unhealthy behavior changes, which could be investigated using similar methods [51].

### Case Exemplar

One compelling case exemplar from our study is Anna (pseudonym), an 80‐90-year-old female who lived alone during the pandemic due to recently becoming widowed. She experienced significant mental and physical decline soon after the lockdown began. She reported “feel[ing] isolated” and increasingly “tired” and “worried” and informed her doctor about feeling short of breath and fatigued. Medical tests were inconclusive. We posit that the clinical team may have benefited from knowing that her sleep duration over 3 months had decreased 1.3%, her sleep restlessness increased 13.9% and efficiency decreased 3.7%, and her time spent out of the home decreased 27.5%—all derived from the digital markers and methods in this study.

Based on these findings and follow-up interviews, the clinical research team determined Anna was likely lonely and needed more social interactions. With her permission, we reached out to community leaders who implemented regular check-ins, including home visits, group walking outdoors, and group puzzling over a web-based platform. Anna responded positively to these interventions, later reporting “feeling better.”

### Integrating Ambient Sensor Information for a Learning Health Care System

A learning health care system could greatly benefit from in-home ambient sensor informatics, which provide insights into the impacts of external events on individuals and populations. Such systems rely on continuously available, objective data to adapt and improve [52]. Smart homes could play a pivotal role by systematically collecting real-time evidence to support clinical decision-making and enhance care effectiveness. By unobtrusively assessing and updating information about patients between care points, the system enables an iterative feedback loop of assessment and intervention, fostering continuous learning and improvement. Additionally, sensor-derived data empower the health care system to address social determinants of health, promoting equitable outcomes for individuals facing external challenges, especially for those already affected by factors like race, gender, age, and income level, which can exacerbate health risks. Adding other opportunities for collecting whole life-space data by including smart watches or other wearables could improve understandings of behavior changes associated with adverse external events. Data from these devices also requires new analytic methods that machine learning is suited to address. Upstream interventions are key to mitigating these risks and improving health equity for these individuals [53]. Objective data and metrics that reveal behavior changes related to external events can help the health care system better address these disparities, allowing targeted individualized care planning based on observed behaviors, ultimately supporting more equitable health outcomes. Sensor-derived information could be used to plan individualized support such as guided indoor exercise programs for older adults unable to leave their home who are also showing less overall daytime activity, or digital mental health support for persons showing increased restless sleep patterns and reporting anxiety, or web-based socialization opportunities to reduce loneliness.

A learning health care system could greatly benefit from in-home ambient sensor informatics, which provide insights into the impacts of external events on individuals and populations. Such systems rely on continuously available, objective data to adapt and improve [52]. Smart homes could play a pivotal role by systematically collecting real-time evidence to support clinical decision-making and enhance care effectiveness. By unobtrusively assessing and updating information about patients between care points, the system enables an iterative feedback loop of assessment and intervention, fostering continuous learning and improvement. Additionally, sensor-derived data empower the health care system to address social determinants of health, promoting equitable outcomes for individuals facing external challenges, especially for those already affected by factors like race, gender, age, and income level, which can exacerbate health risks. Adding other opportunities for collecting whole life-space data by including smart watches or other wearables could improve understandings of behavior changes associated with adverse external events. Data from these devices also require new analytic methods that machine learning is suited to address. Upstream interventions are key to mitigating these risks and improving health equity for these individuals [53]. Objective data and metrics that reveal behavior changes related to external events can help the health care system better address these disparities, allowing targeted individualized care planning based on observed behaviors, ultimately supporting more equitable health outcomes. Sensor-derived information could be used to plan individualized support such as guided indoor exercise programs for older adults unable to leave their home who are also showing less overall daytime activity, or digital mental health support for persons showing increased restless sleep patterns and reporting anxiety, or web-based socialization opportunities to reduce loneliness.

A learning health care system could greatly benefit from in-home ambient sensor informatics, which provide insights into the impacts of external events on individuals and populations. Such systems rely on continuously available, objective data to adapt and improve [52]. Smart homes could play a pivotal role by systematically collecting real-time evidence to support clinical decision-making and enhance care effectiveness. By unobtrusively assessing and updating information about patients between care points, the system enables an iterative feedback loop of assessment and intervention, fostering continuous learning and improvement. Additionally, sensor-derived data empower the health care system to address social determinants of health, promoting equitable outcomes for individuals facing external challenges, especially for those already affected by factors like race, gender, age, and income level, which can exacerbate health risks. Adding other opportunities for collecting whole life-space data by including smart watches or other wearables could improve understandings of behavior changes associated with adverse external events. Data from these devices also requires new analytic methods that machine learning is suited to address. Upstream interventions are key to mitigating these risks and improving health equity for these individuals [53]. Objective data and metrics that reveal behavior changes related to external events can help the health care system better address these disparities, allowing targeted individualized care planning based on observed behaviors, ultimately supporting more equitable health outcomes. Sensor-derived information could be used to plan individualized support such as guided indoor exercise programs for older adults unable to leave their home who are also showing less overall daytime activity, or digital mental health support for persons showing increased restless sleep patterns and reporting anxiety, or web-based socialization opportunities to reduce loneliness.

### Concerns of Older Adults

Privacy, cost, safety, security (data, identity, and health), and reliability are concerns that older adults associate with smart home health monitoring [54-57]. Older adults have indicated they want to be *watched over* but not *watched* [58]. In addition, technologies offering specific health assistance are more desirable than ones that generally monitor and capture data about behaviors and activities unrelated to an older adult’s diagnosis [59,60]. All technologies collecting continuous data aiming to support aging in place require designs that support and embody the ethical principles of autonomy, the right to self-determination, justice, and health equity [61,62].

### Limitations and Future Research

A limitation of this work is the use of a convenience sample of data collected before and during the COVID-19 and wildfire smoke events. Expanding the sample to include greater heterogeneity (race, gender, or socioeconomic status) and representation from more geographic regions would support more generalizable results and potentially identify additional clusters of behavior changes. Sociodemographic factors likely influence behavioral responses to adverse events. Due to the small convenience sample, we were unable to determine the differential impact of sociodemographic factors. Additionally, health data for the case exemplar relied on participant recall, which may be subject to bias and recall error.

This study is further limited by variations in sensor density across participant homes. The number of sensors that were analyzed varied between homes, based on home size and sensor fidelity. While data were normalized to account for these differences, the results could be refined if the numbers were uniform across the sample. We also recognize the inherent limitations of smart home sensors, which capture broad behaviors like navigation patterns and door usage but may miss finer behaviors, such as specific gestures. The confinement of sensors to indoor settings also excludes activities performed outside the home, potentially biasing conclusions. Integrating ambient sensors with wearables and other IoT sources could enhance the breadth and detail of behavior markers. Future research could examine the effects of other external events on older adults’ health, such as migration, economic and policy implications, and the impacts of advances in artificial intelligence.

In this study, we focused on markers that reflect time spent on activities of interest. Future studies may consider additional markers that consider the time of day and location for these markers and integrate new markers into the collection.

### Conclusions

Older adults are increasingly exposed to adverse external events like wildfires. Exposure can lead to behavior changes, putting them doubly at risk. Smart homes offer an innovative solution, affording opportunities for upstream interventions supporting more equitable health outcomes and providing continuous data for the learning health care system. Findings from this study show that the COVID-19 pandemic and the United States Pacific Northwest wildfires impacted community-dwelling older adults’ behaviors with a change in time spent out of the home as the most predictive digital marker, followed by sleep markers, overall activity levels, and the duration of time spent on activities. Findings offer a new type of evidence to support clinical decision-making that considers the context of social determinants of health, like social factors related to the pandemic and exposure to poor air quality.

The rising frequency of external events, combined with the widening gap between available caregivers and the growing population of older adults needing care [63], poses a global gerontological humanitarian challenge. These events disrupt daily routines for older adults, potentially worsening their health and limiting their independence. Smart homes are well-positioned to help bridge this gap by collecting and leveraging in-home ambient sensing data. Further exploration of ambient sensor data integration into clinical decision support tools and the learning health care system is essential. Innovations like these could provide families and health care teams with timely, actionable information that enables person-centered care and supports interventions that promote health equity at scale.

## Abbreviations

GI: Gini impurity
ITS: interrupted time series
